# The Impact of Body Mass Index on the Mortality of Myocardial Infarction Patients With Nonobstructive Coronary Arteries

**DOI:** 10.1002/clc.70013

**Published:** 2024-09-11

**Authors:** Chaohui Dong, Mustafa Kacmaz, Clara Schlettert, Mohammad Abumayyaleh, Ibrahim Akin, Rayyan Hemetsberger, Andreas Mügge, Assem Aweimer, Nazha Hamdani, Ibrahim El‐Battrawy

**Affiliations:** ^1^ Department of Cellular and Translational Physiology Institute of Physiology, Ruhr‐University Bochum Bochum Germany; ^2^ Institut für Forschung und Lehre (IFL), Molecular and Experimental Cardiology Ruhr‐University Bochum Bochum Germany; ^3^ HCEMM‐SU Cardiovascular Comorbidities Research Group, Center for Pharmacology and Drug Research & Development, Department of Pharmacology and Pharmacotherapy Intézet címe Semmelweis University Budapest Hungary; ^4^ Department of Cardiology and Angiology Bergmannsheil University Hospital, Ruhr University of Bochum Bochum Germany; ^5^ First Department of Medicine University Medical Centre Mannheim (UMM) Mannheim Germany; ^6^ Department of Internal Medicine II Division of Cardiology, Medical University of Vienna Vienna Austria; ^7^ Department of Cardiology and Rhythmology University Hospital St. Josef Hospital Bochum, Ruhr University Bochum Bochum Germany; ^8^ Departments of Physiology Cardiovascular Research Institute Maastricht, Maastricht University Maastricht The Netherlands

**Keywords:** adverse events, mortality, myocardial infarction, obesity, overweight

## Abstract

**Objectives:**

Myocardial infarction without significant stenosis or occlusion of the coronary arteries carries a high risk of recurrent major adverse cardiovascular events and poor prognosis. This study aimed to investigate the association between body mass index and outcomes in patients with a suspected myocardial infarction with nonobstructive coronary artery disease (MINOCA).

**Methods:**

Patients were recruited at Bergmannsheil University Hospital from January 2010 to April 2021. The primary outcomes were in‐hospital and long‐term mortality. Secondary outcomes consisted of adverse events during hospitalization and during follow‐up.

**Results:**

A total of 373 patients were included in the study, with a mean follow‐up time of 6.2 years. The patients were divided into different BMI groups: < 25 kg/m² (*n* = 121), 25−30 kg/m² (*n* = 140), and > 30 kg/m² (*n* = 112). In‐hospital mortality was 1.7% versus 2.1% versus 4.5% (*p* = 0.368). However, long‐term mortality tended to be higher in the < 25 kg/m² group compared to the 25−30 and > 30 kg/m² groups (log‐rank *p* = 0.067). Subgroup analysis using Kaplan−Meier analysis showed a higher rate of cardiac cause of death in the < 25 kg/m² group compared to the 25−30 and > 30 kg/m² groups: 5.7% versus 1.1% versus 0.0% (log‐rank *p* = 0.042). No significant differences were observed in other adverse events between the different BMI groups during hospitalization and long‐term follow‐up.

**Conclusions:**

Patients with a BMI < 25 kg/m² who experience a suspected myocardial infarction without significant coronary artery disease may have higher all‐cause mortality and cardiovascular cause of death. However, further data are needed to confirm these findings.

## Graphical abstract

In patients with myocardial infarction without significant coronary artery disease, a body mass index < 25 kg/m^2^ is associated with higher all‐cause mortality and cardiovascular cause of death.

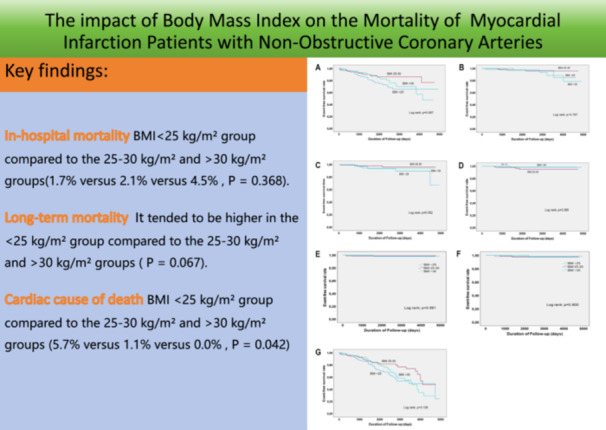

## 1 Introduction

Myocardial infarction with nonobstructive coronary arteries (MINOCA) is a subtype of myocardial infarction (MI) and accounts for approximately 6% (ranging from 5% to 15%) of MI patients who undergo coronary angiography [1, 2, 3, 4]. It was first mentioned nearly 80 years ago [5] and is defined as an acute myocardial infarction (AMI) without a significant coronary artery obstruction on angiography (≥ 50% diameter stenosis in a major epicardial vessel) or specific imaging findings according to the Fourth Universal Definition of MI [6]. Multiple studies have confirmed that MINOCA has a lower mortality rate than acute myocardial infarction with obstructive coronary artery disease (MI‐CAD), but it still carries a high risk of recurrent major adverse cardiovascular events (MACE) and poor prognosis compared to healthy individuals [4, 7, 8]. The prevalence of MINOCA is higher in females and younger individuals [3, 9], and MACE has been associated with cardiovascular risk factors such as smoking, hypertension, and diabetes mellitus [3].

Obesity is a risk factor for metabolic diseases and contributes to atherosclerosis and CAD [10, 11, 12]. The prevalence of overweight and obesity has rapidly increased in recent years [13]. Obesity has been linked to a higher risk of MI and ischemic heart disease, with or without metabolic syndrome [14]. However, the concept of the obesity paradox has emerged in recent years, as several relevant studies have shown that obesity is associated with better cardiovascular disease prognosis compared to individuals of normal weight [15, 16, 17]. Additionally, a recent study found that normal‐weight individuals with diabetes had comparable adjusted cardiovascular mortality to obese individuals without diabetes [18]. Another study revealed that patients with a body mass index (BMI) less than 18.5 kg/m^2^ had higher mortality rates than both normal weight and obese patients with AMI [19].

While myocardial infarction is a fatal disease and patients suffer from acute chest pain and persistent ECG changes associated with elevated troponins and in a coronary angiography a culprit lesion is confirmed. MINOCA is a syndrome with multiple etiologies from cardiac and noncardiac causes and is commonly seen in hospitalized patients diagnosed with MI. Therefore, an accurate and systematic examination is needed to help identify the cause of MINOCA in different patients, stratify the risk and implement the most appropriate treatment. Patients with MINOCA, especially those with normal coronary angiography, are often misdiagnosed as “non‐cardiac patients,” missing the optimal treatment opportunity and affecting patient prognosis. Most studies concentrated on the association between obesity and myocardial infarction and confirmed that obesity should be an important risk factor. MINOCA is an important subtype of which has a different mechanism. With the increasing diagnosis of MINOCA, there is no relevant data analysis on whether obesity is related to it. And no relevant studies have focused on the association between BMI and prognosis in patients with MINOCA. Therefore, the present study aims to evaluate the association between BMI and the clinical presentation and outcomes of patients with suspected MINOCA in a large cohort.

## 2 Methods and Materials

### 2.1 Study Population

From January 2010 to April 2021, a total of 24 775 patients who underwent coronary angiography at Bergmannsheil University Hospital were enrolled. All participants aged ≥ 18 years old were eligible for the study, and those with incomplete information (e.g., missing ventilation protocols) were excluded. The study protocol received approval from the Ethics Committee of the Medical Faculty of Ruhr University Bochum (22‐7684). The informed consent was obtained from each patient and the study protocol conforms to the ethical guidelines of the 1975 Declaration of Helsinki.

### 2.2 Data Collection

Three hundred and seventy‐three had elevated troponin values without significant coronary artery stenosis confirmed by coronary angiography. Cardiac troponin levels needed to be elevated, with at least one value surpassing the 99th percentile. All demographic information and cardiovascular risk factors were collected from medical records. Coronary angiography results, and treatment approach in the hospital were documented during the hospitalization period. We also collected medication information used at the time of admission and discharge.

### 2.3 Definitions and Outcomes

The inclusion based on assessments of laboratory reports, ECGs, and angiograms. The BMI was calculated as weight in kilograms divided by the square of height in meters. Overweight was defined as a BMI between 25.0 and 30.0 kg/m^2^, while obesity was defined as a BMI > 30.0 kg/m^2^ according to guidelines [20].

The major adverse events in the hospital were defined as a combination of cardiopulmonary resuscitation, left ventricular thrombus, thromboembolic events, pulmonary edema, cardiogenic shock, invasive and noninvasive ventilation, stroke, life‐threatening cardiac arrhythmias, supraventricular arrhythmias, and overall mortality. We also divided in‐hospital death into cardiac‐caused and noncardiac‐caused as far as the cause of death was found. During the follow‐up period, long‐term outcomes (including stroke, thromboembolic events, recurrence of MINOCA, cardiac arrest, percutaneous coronary intervention [PCI], and all‐cause mortality) were documented. And we also recorded cardiac‐caused death and noncardiac‐caused death.

The primary outcomes were defined as in‐hospital and long‐term mortality. The second outcomes were other adverse outcomes.

### 2.4 Statistics Analysis

Mean ± standard deviation was used in the continuous variables with normal distribution, while median (interquartile range) was used in the continuous variables with non‐normal distribution. Differences in demographic and cardiovascular risk factors between different BMI groups were tested using the Students *t*‐test, chi‐square test, or analysis of variance. The cumulative incidence of adverse events among the groups was shown by Kaplan−Meier analysis and compared using the log‐rank test. Statistically significant was declared if two‐sided *p* < 0.05, and data were analyzed using SPSS Statistics 29.0 software.

## 3 Results

### 3.1 Basic Characteristics

**Figure 1:**
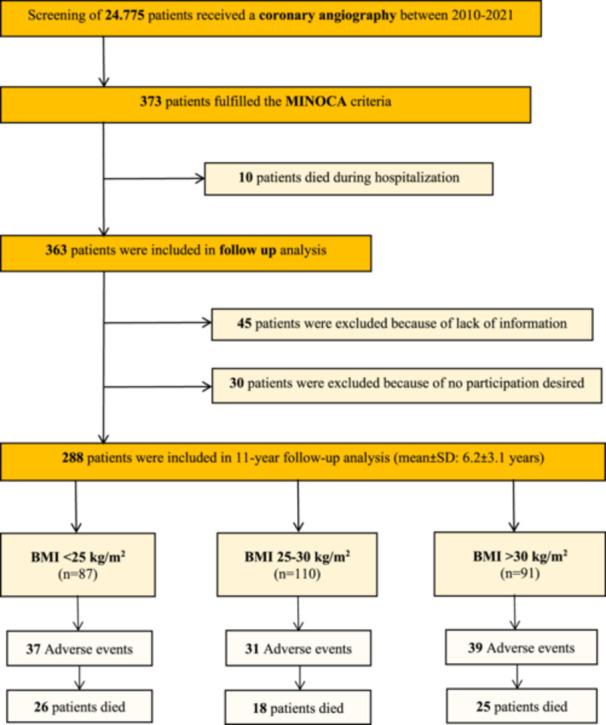
Flowchart presenting the screened data and included patients for the present study.

A total of 373 patients were enrolled in the study, and the mean follow‐up time was 6.2 years. The flowchart is shown in Figure 1. The average age of the patients was 63 ± 15.6 years old, and the proportion of females was 50.4%. Among the patients, 121 (32.4%) were in the BMI < 25 kg/m² group, 140 (37.5%) were overweight (BMI 25−30 kg/m²), and 112 (30.0%) were obese (BMI > 30 kg/m²). There were no significant differences in age between the groups, but the BMI < 25 kg/m² group had a higher proportion of females (*p *< 0.05, Table 1).

**Table 1:** Baseline characteristics of 373 patients initially presenting MINOCA according to BMI.

Variables	All patients *n* = 373	< 25 *n* = 121	25−30 *n* = 140	> 30 *n* = 112	*p* value
**A**ge—years, mean ± SD	63 ± 15.6	62.5 ± 17	62.5 ± 17	63 ± 14	0.839
Male—*n* (%)	185 (49.6)	48 (39.7)	78 (55.7)	59 (52.7)	0.026
Duration of hospitalization—days, mean ± SD	10 ± 8.5	9 ± 6	9 ± 6	10.1 ± 7	0.009
Symptoms—*n* (%)					
Angina pectoris	226 (61.6)	76 (64.4)	82 (59.4)	68 (61.3)	0.715
Dyspnea	164 (44.4)	55 (46.6)	50 (35.7)	59 (53.2)	0.019
Palpations	45 (12.3)	13 (11)	21 (15.2)	11 (9.9)	0.396
Clinic parameter, mean ± SD					
Systolic BP, mmHg	146.6 ± 64.4	153.5 ± 98	153.5 ± 98	147.3 ± 29	0.235
Diastolic BP, mmHg	84.8 ± 18.1	82.9 ± 18	82.9 ± 18	87.2 ± 19	0.268
Heart rate, bpm	89.8 ± 28.6	87 ± 26	86.8 ± 26	95.6 ± 31	0.041
ECG—*n* (%)					
ST elevation	55 (14.8)	23 (19)	14 (10)	18 (16.1)	0.111
Inversed T‐waves	183 (49.2)	67 (55.8)	70 (50)	46 (41.1)	0.078
Medical history—*n* (%)					
Current smoking	85 (23.1)	23 (19.2)	38 (27.3)	24 (22)	0.285
Arterial hypertension	253 (68.2)	74 (61.2)	96 (68.6)	83 (75.5)	0.066
Dyslipidemia	99 (26.6)	23 (19)	41 (29.3)	35 (31.5)	0.065
Diabetes mellitus	65 (17.5)	10 (8.3)	18 (12.9)	37 (33.3)	< 0.001
COPD	47 (12.6)	25 (20.7)	10 (7.1)	12 (10.8)	0.004
Bronchial asthma	33 (8.9)	5 (4.1)	18 (12.9)	10 (9)	0.047
Malignancy	47 (12.7)	19 (15.8)	16 (11.4)	12 (10.9)	0.455
Kidney disease	53 (14.3)	15 (12.4)	17 (12.1)	21 (18.9)	0.244
Neurological disease	90 (24.3)	40 (33.1)	24 (17.1)	26 (23.6)	0.011
Autoimmune disease	17 (4.6)	5 (4.1)	7 (5)	5 (4.6)	0.946
Psychiatric disease	39 (10.5)	8 (6.6)	19 (13.6)	12 (10.8)	0.187
Pacemaker	14 (3.8)	4 (3.3)	7 (5)	3 (2.7)	0.613
Supraventricular arrhythmias^a^	57 (15.4)	23 (19.2)	21 (15)	13 (11.8)	0.316
Atrial fibrillation	57 (15.4)	23 (19.2)	21 (15)	13 (11.8)	0.316
Atrial flutter	0 (0)	0 (0)	0 (0)	0 (0)	0
Laboratory values, mean ± SD					
Troponin (µg/L)	2.1 ± 8.4	1.6 ± 5.5	1.3 ± 7	2.6 ± 9.8	0.435
Creatin phosphatkinase (U/L)	288.6 ± 548.9	282.5 ± 501	260.1 ± 451	330.6 ± 692	0.594
BNP (pg/mL)	433.5 ± 1004.5	598 ± 1271	378.3 ± 1056	323.5 ± 479	0.187
Creatinine (mg/dL)	3.3 ± 30.9	5.7 ± 51.7	1.1 ± 0.7	3.5 ± 17	0.483
Echocardiography data, *n* (%)					
LVEF % (on admission), mean ± SD	36.9 ± 25.7	37.9 ± 26.4	37.6 ± 25.4	34.93 ± 25	0.621
LVEF ≥ 50%	170 (66.9)	54 (64.3)	69 (72.6)	47 (62.7)	0.323
LVEF 49%−40%	31 (12.2)	14 (16.7)	8 (8.4)	9 (12)	0.245
LVEF < 40%	52 (20.5)	15 (17.9)	18 (19)	19 (25.3)	0.458
Left ventricular hypertrophy	103 (29.1)	33 (28.7)	30 (22.7)	40 (37.4)	0.046
Tricuspid valve regurgitation	89 (24.2)	28 (23.5)	39 (28.1)	22 (20)	0.332
‐ Mild	63 (17.1)	19 (16)	28 (20.2)	16 (14.6)	0.470
‐ Moderate	23 (6.3)	8 (6.7)	10 (7.2)	5 (4.6)	0.671
‐ Severe	3 (0.8)	1 (0.8)	1 (0.7)	1 (0.9)	0.986
Mitral valve regurgitation	106 (28.8)	33 (27.7)	43 (30.9)	30 (27.3)	0.780
‐ Mild	77 (20.9)	22 (18.3)	32 (23)	23 (20.9)	0.653
‐ Moderate	19 (5.2)	9 (7.5)	5 (3.6)	5 (4.6)	0.347
‐ Severe	10 (2.7)	2 (1.7)	6 (4.3)	2 (1.8)	0.337
Aortic valve regurgitation	39 (10.6)	14 (11.8)	16 (11.5)	9 (8.2)	0.617
‐ Mild	31 (8.4)	11 (9.2)	13 (9.4)	7 (6.4)	0.651
‐ Moderate	4 (1.1)	1 (0.8)	1 (0.7)	2 (1.8)	0.676
‐ Severe	4 (1.1)	2 (1.7)	2 (1.4)	0 (0)	0.417
Drugs on admission, *n* (%)					
β‐Blocker	131 (35.2)	47 (38.8)	40 (28.8)	44 (39.3)	0.134
ACE inhibitor	121 (32.6)	44 (36.4)	35 (25.4)	42 (37.5)	0.071
Sartane	57 (15.3)	12 (9.9)	27 (19.4)	18 (16.1)	0.102
Ca‐Blocker	74 (19.9)	22 (18.2)	24 (17.3)	28 (25)	0.266
Diuretics	101 (27.2)	29 (24)	32 (23)	40 (35.7)	0.051
Anticoagulants^b^	58 (15.6)	27 (22.3)	18 (13)	13 (11.6)	0.044
Aspirin	79 (21.2)	31 (25.6)	24 (17.3)	24 (21.4)	0.260
Clopidogrel	18 (4.8)	9 (7.4)	3 (2.2)	6 (5.4)	0.135
Prasugrel	0 (0)	0 (0)	0 (0)	0 (0)	0
Antiarrhythmics^c^	10 (2.7)	2 (1.7)	5 (3.6)	3 (2.7)	0.629

Abbreviations: ACE, angiotensin‐converting‐enzyme; AV, atrioventricular; BMI, body mass index; BNP, brain natriuretic peptide; BP, blood pressure; COPD, chronic obstructive pulmonary disease; ECG, electrocardiogram; HFmrEF, heart failure with mid range ejection fraction; HFpEF, heart failure with preserved ejection fraction; HFrEF, heart failure with reduced ejection fraction; LV EF, ejection fraction; MINOCA myocardial infarction with nonobstructive coronary artery disease; SD, standard deviation.

a Only one malignant cardiac/supraventricular arrhythmia is counted per patient (even if one patient has several arrhythmias at the same time).

b Cumarine, heparin, selective factor 10‐blocker, direct thrombin inhibitors.

c Ivabradine, flecainid, sotalol, dronedaron, digitalis.

*p* values for the comparison between groups of ages.

Among the patients included in the registry, the results indicated that the obesity (BMI > 30 kg/m²) group had the longest hospitalization days and the highest proportion of dyspnea (*p* < 0.05). Additionally, the heart rate was higher in the BMI > 30 kg/m² group (95.6 ± 31 bpm) compared to the other groups (BMI 25−30 kg/m² 86.8 ± 26 bpm and < 25 kg/m² 87 ± 26 bpm; *p* = 0.04).

Regarding medical histories, the obesity group had the highest proportion of diabetes mellitus, while the BMI < 25 kg/m² group had the highest proportion of neurological diseases and chronic obstructive pulmonary disease (COPD) (*p* < 0.05). There were no significant differences in the other medical histories. All patients enrolled had an echocardiography in the hospital, and the results suggested that the obesity group had the most severe left ventricular hypertrophy (*p* < 0.05). The left ventricular ejection fraction was similar in all groups. Laboratory values such as troponin, creatinine kinase, and BNP were similar in all groups. Drugs at discharge were similar in all groups (Supporting Information S1: Table 1).

### 3.2 Primary Outcomes

#### 3.2.1 Association Between BMI and In‐Hospital Mortality and Long‐Term Mortality

In‐hospital death occurred in 10 (2.7%) patients, 6 (1.6%) due to cardiac causes and 4 (1.7%) due to noncardiac causes (Table 2). The in‐hospital mortality rates were as follows: 2 (1.7%) in the BMI < 25 kg/m² group, 3 (2.1%) in the BMI 25−30 kg/m² group, and 5 (4.5%) in the BMI > 30 kg/m² group; *p* = 0.368 (Supporting Information S1: Figure 1). During the follow‐up period, 69 (24.1%) patients died. The rates of all‐cause mortality were 29.9% in the BMI < 25 kg/m² group, 16.7% in the BMI 25−30 kg/m² group, and 27.5% in the BMI > 30 kg/m² group; *p*= 0.067. Subgroup analysis using Kaplan−Meier analysis revealed a weak correlation between the obesity group and an increased risk of cardiac death (log‐rank *p*= 0.042; Table 3). No significant differences were observed in noncardiac mortality rates.

**Table 2:** In‐hospital complications according to BMI.

	All patients *n* = 373	< 25 *n* = 121	25−30 *n* = 140	> 30 *n* = 112	*p* value
Adverse event	132 (35.5)	39 (32.2)	50 (35.7)	43 (38.4)	0.616
CPR	7 (1.9)	1 (0.8)	4 (2.9)	2 (1.8)	0.484
Left ventricular thrombus	3 (0.8)	0 (0)	2 (1.4)	1 (0.9)	0.435
Thromboembolic event	3 (0.8)	1 (0.8)	2 (1.4)	0 (0)	0.453
Pulmonary edema	9 (2.7)	3 (2.5)	3 (2.1)	3 (2.7)	0.961
Cardiogenic shock	9 (2.7)	3 (2.5)	3 (2.1)	3 (2.7)	0.961
Invasive ventilation	29 (7.8)	7 (5.8)	11 (7.9)	11 (9.8)	0.518
Noninvasive ventilation	11 (3)	4 (3.3)	3 (2.1)	4 (3.6)	0.771
Stroke	1 (0.3)	0 (0)	1 (0.7)	0 (0)	0.436
Malignant cardiac arrhythmias (on admission/in hospital)	39 (10.5)	9 (7.4)	13 (9.3)	17 (15.2)	0.133
Bradycardiac arrhythmias	13 (3.5)	4 (3.3)	3 (2.1)	6 (5.4)	0.383
‐ AV block 2 Mobitz	3 (0.8)	0 (0)	2 (1.4)	1 (0.9)	0.435
‐ AV block 3	1 (0.3)	0 (0)	1 (0.7)	0 (0)	0.436
‐ Asystole	10 (2.7)	4 (3.3)	1 (0.7)	5 (4.5)	0.164
Ventricular arrhythmias	14 (3.8)	3 (2.5)	5 (3.6)	6 (5.4)	0.510
‐ Sustained	8 (2.2)	3 (2.5)	1 (0.7)	4 (3.6)	0.290
‐ Non‐sustained	6 (1.6)	0 (0)	3 (2.1)	3 (2.7)	0.291
‐ Ventricular fibrillation	16 (4.3)	3 (2.5)	7 (5)	6 (5.4)	0.487
Torsades de pointes	2 (0.5)	1 (0.8)	1 (0.7)	0 (0)	0.650
Supraventricular arrhythmias	85 (22.8)	30 (24.8)	32 (22.9)	23 (20.5)	0.742
Atrial fibrillation	77 (20.6)	28 (23.1)	27 (19.3)	22 (19.6)	0.711
‐ First appearance	43 (11.5)	13 (10.7)	16 (11.4)	14 (12.5)	0.915
‐ Recurrence	34 (9.1)	15 (12.4)	11 (7.9)	8 (7.1)	0.308
Atrial flutter	9 (2.4)	2 (1.7)	5 (3.6)	2 (1.8)	0.529
‐ First appearance	9 (2.4)	2 (1.7)	5 (3.6)	2 (1.8)	0.529
‐ Recurrence	0 (0)	0 (0)	0 (0)	0 (0)	—
In‐hospital death	10 (2.7)	2 (1.7)	3 (2.1)	5 (4.5)	0.368
Cardiac caused death	6 (1.6)	1 (0.8)	3 (2.1)	2 (1.8)	0.692
Noncardiac caused death	4 (1.1)	1 (0.8)	0 (0)	3 (2.7)	0.116

Abbreviations: Adverse event, major adverse cardiac and cerebrovascular events; CPR, cardiopulmonary resuscitation; ECMO, extracorporal.

**Table 3:** Extra‐hospital complications (during follow‐up) according to BMI.

	All patients *n* = 288	< 25 *n* = 87	25−30 *n* = 110	> 30 *n* = 91	*p* value
Adverse event	100 (34.8)	34 (39.1)	30 (27.5)	36 (39.6)	0.126
Stroke	10 (4.2)	3 (4.4)	3 (3.2)	4 (5.3)	0.797
Thromboembolic event	6 (2.5)	1 (1.4)	4 (4.3)	1 (1.4)	0.385
Recurrence of troponin‐positive with nonobstructive CAD	3 (1.3)	1 (1.4)	1 (1.1)	1 (1.4)	0.981
Percutaneous coronary intervention	17 (7.2)	7 (10)	4 (4.3)	6 (8.2)	0.352
Death	69 (24.1)	26 (29.9)	18 (16.7)	25 (27.5)	0.067
‐ Cardiac caused death	5 (2.1)	4 (5.7)	1 (1.1)	0 (0)	0.042
‐ Noncardiac caused death	12 (5.1)	5 (7.1)	1 (1.1)	6 (8.3)	0.075

Abbreviations: Adverse event, major adverse cardiac and cerebrovascular events; CAD, coronary artery disease; CPR, cardiopulmonary resuscitation; NSTEMI, non‐ST‐segment elevation myocardial infarction; STEMI, ST‐segment elevation myocardial infarction.

### 3.3 Second Outcomes

#### 3.3.1 Association Between BMI and Other Adverse Events In‐Hospital and Long‐Term Follow‐Up

As shown in Supporting Information S1: Figure 1 and Table 2, there were no significant differences observed between the different BMI groups and the occurrence of adverse in‐hospital events. These events included a combination of cardiopulmonary resuscitation, left ventricular thrombus, thromboembolic events, pulmonary edema, cardiogenic shock, invasive and noninvasive ventilation, stroke, life‐threatening cardiac arrhythmias, and supraventricular arrhythmias.

**Figure 2:**
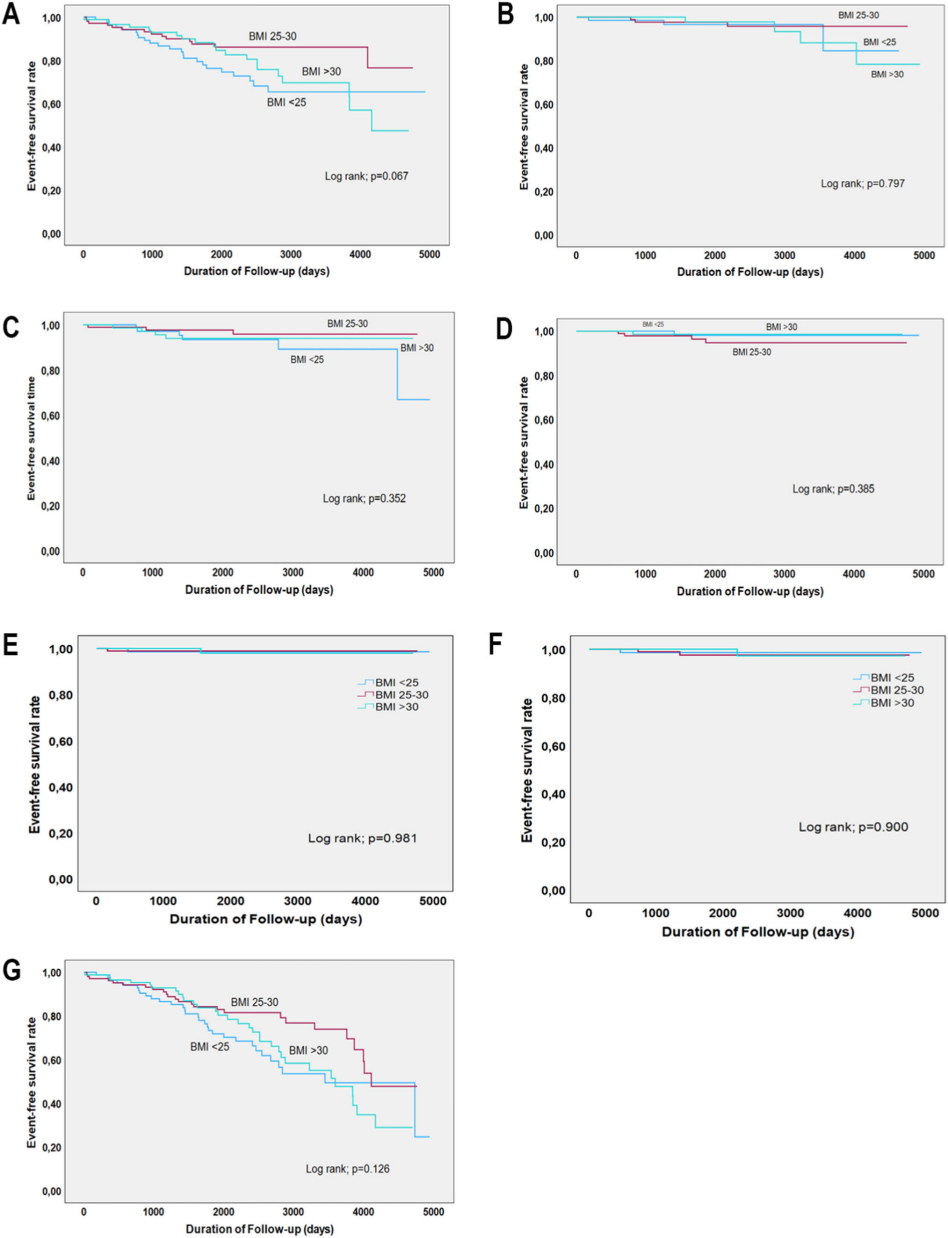
The Kaplan−Meier (KM) curves between adverse events according to different BMI ranges. (A) Shows the KM curve of different BMI ranges and mortality; (B) shows the KM curve of BMI ranges and stroke; (C) shows the KM curve of BMI ranges and percutaneous coronary intervention; (D) shows the KM curve of BMI ranges and thromboembolic events; (E) shows the KM curve of BMI ranges and recurrence of MINOCA; (F) shows the KM curve of BMI ranges and cardiac arrest; and (G) shows the KM curve of BMI ranges and all adverse events.

During the follow‐up period, the Kaplan−Meier curves indicated that there was no significant correlation found between the occurrence of adverse events (such as stroke, thromboembolic events, recurrence of MINOCA, cardiac arrest, and PCI) among all the groups (Figure 2).

We also did an extra analysis based on anticoagulant therapy, and the results indicated that during the long‐term follow‐up, the occurrence of adverse events and death were not significantly different between the novel oral anticoagulants (NOACs) treatment group and non‐NOACs treatment group (Supporting Information S1: Table 2).

## 4 Discussion

In this study, we examined the relationship between BMI and clinical outcomes in patients suspected of MINOCA. Our results indicate that there were no significant differences in adverse events between BMI groups both during hospitalization and in the long‐term. However, when we conducted a subgroup analysis, we found that patients in the obesity group had the lowest prevalence of all‐cause death and cardiac‐caused death.

MINOCA is a cardiovascular disease that poses a high risk for cardiac morbidity and mortality. The prevalence of MINOCA is higher in females and younger people [3, 9]. Consistent with other studies, we found that MINOCA patients tend to be younger. In our study, the enrolled patients were overweight (37.5%) or obese (30%), which was higher than in previous studies [7, 21]. However, the VIRGO study reported an even higher proportion of obesity, up to 42.1% [3], which exceeded our findings. Another recent study involving 233 MINOCA patients showed an average duration of hospitalization [22] of 5 days, whereas our study indicated a duration of 10 days, with the obesity group having the longest hospitalization. Obesity is often accompanied by metabolic diseases [23], and our study showed that patients in the obesity group had a higher proportion of diabetes, higher heart rates, and more symptoms such as dyspnea. These factors may contribute to the longer duration of hospitalization observed in the obesity group compared to other groups.

The in‐hospital mortality rate in our study was 2.7%, with the highest rate observed in the obesity group, although it was lower than in a previous study [24]. However, no significant differences were found between different BMI groups. Previous studies have reported 1‐year mortality rates ranging from 1.1% to 12.5% for MINOCA patients, and a study involving 9092 patients with MINOCA reported a mortality rate of 14% during a mean follow‐up of 4.5 years. In our study, during a mean follow‐up of 6.2 years, 24.1% of the MINOCA patients died. No significant differences were observed between different BMI groups, but the all‐cause mortality and cardiac‐caused death were higher in patients with a BMI < 25 kg/m² compared to those in the overweight and obesity groups. Obesity is often accompanied by metabolic diseases and may increase the incidence of cardiovascular diseases. A pooled analysis of 97 prospective cohorts with 1.8 million participants indicated that each 5 kg/m² increase in BMI was associated with an increased risk ratio of 1.27 for coronary heart disease and 1.18 for stroke [25]. Other studies have reported the obesity paradox, which suggests that overweight and obesity may be associated with a more favorable prognosis for cardiovascular and cerebrovascular diseases [15, 26]. The mechanisms underlying the higher event rate in the BMI 25−30 kg/m² range in MINOCA patients remain unclear, but there are several potential reasons. Non‐purposeful weight loss, greater metabolic reserves, less cachexia, and protective cytokines may all contribute to a better prognosis for cardiovascular events [27]. Some other confounding factors such as dietary habits, physical activity levels, and socioeconomic status were not available in our study, these important factors may also have an effect on the association on BMI and MINOCA.

The adverse outcomes of MINOCA varied depending on the duration of follow‐up in different studies. Previous studies have shown a prevalence of MACE ranging from 4.6% to 23.9% [2, 4, 28], which is lower than what we found in our study. This difference may be due to variations in follow‐up time and inclusion criteria. One study found that 6.3% of MINOCA patients were hospitalized due to recurrent MI over an average follow‐up period of 17 months. Of these patients, 53% had no obstructive CAD and 47% had obstructive CAD [29]. In our study, we observed that during a mean follow‐up of 6.2 years, 1.3% of people were hospitalized due to a recurrence of MI, and 7.2% of patients underwent percutaneous coronary artery intervention. No significant differences were found in terms of in‐hospital or long‐term adverse events among different BMI groups. However, a recent study including 281 patients reported that MINOCA patients with a BMI greater than 25 kg/m^2^ had a higher prevalence of MACE over a mean follow‐up of 28 months [21], which contradicts our findings. Another study reported that BMI was not associated with MACE [9], although it is worth noting that only one patient in this cohort had a BMI greater than 30 kg/m^2^.

Our study found that adverse events are comparable over follow‐up in patients treated with NOAC at discharge compared to no NOAC treatment. Due to the limited number of patients, we could not further analyze by BMI grouping, and the number of endpoint events was too small, further studies may be needed to determine the role of anticoagulation therapy in patients with MINOCA.

## 5 Study Limitations

This monocenter study is important for understanding the relationship between BMI and MINOCA. However, there are several limitations. First, the cross‐sectional design cannot determine causality. Second, the cause of death in many deceased cases during the follow‐up period remained unknown. Third, the definition of MINOCA was solely based on BMI and did not include information on body fat composition, epicardial fat, or muscle mass. Fourth, the small number of endpoints may have influenced the results. Lastly, the monocentric cross‐sectional design with a low number of patients limits the ability to establish causality between BMI and long‐term outcomes in MINOCA patients. Longitudinal studies would provide stronger evidence for causal relationships. Therefore, the conclusions obtained need to be verified by more multicenter studies.

## 6 Conclusions

In summary, our study revealed an association between BMI and MINOCA. There were no statistically significant differences between overweight, obesity, hospitalization, and the long‐term outcomes of MINOCA. However, in terms of long‐term death and cardiac death, it seems that individuals with a BMI of 25−30 kg/m² had lower mortality and cardiac mortality rates. This finding requires further research to confirm.

## Author Contributions

Ibrahim El‐Battrawy and Nazha Hamdani put forwards the design and conception of this study. Mustafa Kacmaz and Clara Schlettert completed data collection. Clara Schlettert participated in the data analysis, and Chaohui Dong completed the manuscript writing. Mohammad Abumayyaleh, Ibrahim Akin, Rayyan Hemetsberger, Andreas Mügge, and Assem Aweimer gave some important suggestions on article revision. All authors have read and agreed to the published vision of the manuscript.

## Ethics Statement

The study protocol received approval from the Ethics Committee of the Medical Faculty of Ruhr University Bochum.

## Conflicts of Interest

The authors declare no conflicts of interest.

## Supporting information

Supporting information.

## Data Availability

The data can be obtained by contacting the corresponding author. All authors take responsibility for all aspects of the reliability and freedom from bias of the data presented and their discussed interpretation.
