# Supplementary material for: The Impact of Body Mass Index on the Mortality of Myocardial Infarction Patients With Nonobstructive Coronary Arteries
Source: Clin Cardiol. 2024 Sep 11;47(9):e70013. doi: 10.1002/clc.70013 (PMC11390790; doi:10.1002/clc.70013)
Supplement: Supplementary file 1 — Supporting information. [file CLC-47-e70013-s001.docx]

| **sTable 1:** Medication at discharge | | | | | |
| --- | --- | --- | --- | --- | --- |
|  | **All patients**  **n=373** | **<25**  **n=121** | **25-30**  **n=140** | **>30**  **n=112** | **P**  **Value** |
| ß-Blocker | 270 (72.4) | 84 (70) | 99 (70.7) | 87 (78.4) | 0.319 |
| ACE inhibitor | 224 (60.1) | 74 (61.7) | 77 (55) | 73 (65.8) | 0.251 |
| Sartane | 62 (16.6) | 13 (10.8) | 27 (19.3) | 22 (19.8) | 0.107 |
| Ca Blocker | 100 (26.8) | 31 (25.8) | 30 (21.4) | 39 (35.1) | 0.055 |
| Diuretics | 166 (44.5) | 53 (43.8) | 55 (39.3) | 58 (51.8) | 0.138 |
| Anticoagulants* | 104 (27.9) | 39 (32.5) | 37 (26.4) | 28 (25.2) | 0.419 |
| Aspirin | 175 (46.9) | 55 (45.8) | 62 (44.3) | 58 (52.3) | 0.461 |
| Clopidogrel | 68 (18.2) | 20 (16.5) | 26 (18.6) | 22 (19.6) | 0.822 |
| Prasugrel | 0 (0) | 0 (0) | 0 (0) | 0 (0) | 0 |
| Antiarrhythmics** | 26 (7) | 8 (6.7) | 10 (7.1) | 8 (7.2) | 0.983 |
| **ACE**, Angiotensin-converting-enzyme; * Cumarine, Heparin, selective factor 10-blocker, direct thrombin inhibitors; ** Ivabradin, Flecainid, Sotalol, Dronedaron, Digitalis | | | | | |

| sTable 2: Extra-hospital complications (during follow up) according to NOACs | | | | |
| --- | --- | --- | --- | --- |
|  | **All patients**  **n=242** | **NOACs**  **n=64** | **Non-NOACs**  **n=178** | **P Value** |
| Adverse event | 66 (27.3) | 21 (32.8) | 45 (25.3) | 0.246 |
| Death | 37 (15.3) | 10 (15.6) | 27 (15.2) | 0.894 |
| - cardiac caused death | 3 (1.2) | 2 (3.1) | 1 (0.6) | 0.117 |
| - non-cardiac caused death | 9 (3.7) | 3 (4.7) | 6 (3.4) | 0.654 |
| NOAC, new oral anticoagulants; adverse event, major adverse cardiac and cerebrovascular events; CAD, coronary artery disease | | | | |

**sFigure 1: Intra-hospital events related to BMI.**
